# Postabortion Care in Humanitarian Emergencies: Improving Treatment and Reducing Recurrence

**DOI:** 10.9745/GHSP-D-18-00400

**Published:** 2019-08-22

**Authors:** Meghan Gallagher, Catherine Morris, Mariam Aldogani, Claire Eldred, Abdikani Hirsi Shire, Emily Monaghan, Sarah Ashraf, Janet Meyers, Ribka Amsalu

**Affiliations:** a Save the Children, Washington, DC, USA.; b Save the Children, Hodeida, Yemen.; c Save the Children, Goma, Democratic Republic of the Congo.; d Save the Children, Puntland, Somalia.; e Save the Children, London, United Kingdom

## Abstract

**Background:**

Unsafe abortion contributes to maternal mortality worldwide and disproportionately affects the most disadvantaged women and girls; thus, improving the treatment of complications of abortion is essential. Shifting PAC treatment from sharp dilation and curettage (D&C) to the use of aspiration techniques, notably manual vacuum aspiration (MVA), and medical treatment with misoprostol improves health outcomes. Equally critical is ensuring that women have access to voluntary contraception after an abortion to prevent future unintended pregnancies. In humanitarian settings, access to voluntary family planning to disrupt the cycle of unsafe abortion is even more critical because access to quality services cannot be guaranteed due to security risks, migration, and devastation of infrastructure. Save the Children applied a multipronged postabortion care (PAC) approach in the Democratic Republic of the Congo (DRC), Somalia, and Yemen that focused on capacity building; assurance of supplies and infrastructure; community collaboration and mobilization; and monitoring and evaluation.

**Methods:**

Program-level data were extracted for each of the 3 countries from the inception of their program through 2017. The sources of information included monthly service delivery reports that tracked key PAC indicators as well as qualitative data from evaluations of community mobilization activities.

**Results:**

The number of PAC clients increased in all countries. In the DRC in 2012, 19% of PAC clients requiring treatment received D&C; in 2017 the percentage was reduced to 3%. In 2013, 25% of all PAC clients in Yemen were treated with D&C; this percentage was reduced to 3% in 2017. The proportion of women choosing contraception after an abortion increased. In 2012, only 42% of all PAC clients in the DRC chose a contraceptive method; by 2017, the proportion had increased to 70%. Somalia had substantial increases in PAC demand, with the percentage of all PAC clients electing contraception increasing from 64% in 2012 to 82% in 2017. In Yemen, where the health system has been constrained due to severe conflict, the percentage of PAC clients choosing voluntary contraception rose from 17% in 2013 to 38% in 2017. Uptake and demand for PAC was mobilized through targeted community outreach in each context.

**Conclusion:**

These data demonstrate that providers can effectively shift away from D&C as treatment for PAC and that contraceptive uptake by PAC clients can increase substantially, even in settings where the use of contraception after abortion is often stigmatized.

## Abstract (precis)

Despite the challenging environment of humanitarian emergencies, with focused programmatic attention, demand for quality postabortion care can be created and services delivered while voluntary contraceptive uptake for PAC clients can simultaneously increase substantially, even in settings where the use of contraception after abortion is often stigmatized. Greater representation of long-acting methods, as a proportion of the methods PAC clients chose, occurred in all 3 countries’ method mix, but at different rates.

## BACKGROUND

An estimated 25 million unsafe abortions occur globally each year, with 97% of these procedures occurring in developing countries.^1^ Between 4.7% and 13.2% of maternal deaths worldwide are attributable to unsafe abortion, with sub-Saharan Africa and southern Asia disproportionately accounting for 83.8% of this mortality.^2^ In the developing world, nearly 7 million women and girls present in health facilities to receive treatment for complications due to unsafe abortion annually, while many, who are often the most disadvantaged, forgo care completely.^3^ Analyses of surveys from 14 countries with recent abortion incidence studies found that going without needed care is more common among poor women living in rural areas than women who are not poor and live in urban areas; approximately 49% of the rural poor who need care due to complications do not obtain it, while 21% of the nonpoor urban bypass care.^4^ Although estimates of unsafe abortion are not available in the context of humanitarian crises, the need for quality postabortion care (PAC) likely increases given the deterioration of the health system and the consequent decreased access to emergency obstetric services and safe delivery.^5^^–^^7^

To reduce maternal morbidity and mortality in developing countries, improving the treatment of abortion complications is essential. As per World Health Organization (WHO) recommendations, shifting from sharp dilation and curettage (D&C) to the use of aspiration techniques, notably manual vacuum aspiration (MVA) and medical treatment with misoprostol for PAC, improves health outcomes and expands the availability of services through task shifting.^8^ In addition to improving safety, the shift away from D&C reduces the length of in-patient hospital stays, reduces the time required for recovery, and is more cost-effective overall.^9^^,^^10^

To reduce maternal morbidity and mortality in developing countries, improving the treatment of abortion complications is essential.

It is equally critical to prevent future unintended pregnancies by ensuring that women have access to voluntary contraception after an abortion. Although existing evidence suggests that the majority of PAC clients are interested in adopting a method of contraception, uptake varies from 25% to 77% due to differences in service delivery, human resources, and commodity security.^11^^–^^14^ The high unmet need for contraception among PAC clients combined with a nearly immediate return to fertility after an abortion contributes to the higher likelihood of subsequent unintended pregnancies and abortions among women who have previously had an abortion.^11^^,^^15^^,^^16^ Although postabortion contraceptive counseling and provision of voluntary contraception is an essential element of all PAC models, PAC has historically focused on treating immediate life-threatening symptoms such as hemorrhage and sepsis rather than addressing women's desire to delay, space, or limit future childbearing. Therefore, they miss an opportunity to disrupt the cycle of repeat unintended pregnancy that leads to further instances of unsafe abortion.^17^^,^^18^ Successful postabortion contraceptive programs have shown that offering a broad mix of methods on site improves overall uptake.^12^^,^^19^

In humanitarian settings, disrupting the cycle of unsafe abortion is even more critical because it is sometimes difficult to ensure access to quality PAC due to security risks, forced migration, and devastation of infrastructure.^20^^,^^21^ Further, a broad contraceptive method mix, including the availability of long-acting reversible contraceptives (LARCs), for PAC clients allows women to avert future unintended pregnancies, choose a method that suits their lifestyle, and, in the case of LARCs, have longer periods of more effective contraceptive coverage when desired. The Minimum Initial Service Package (MISP) for reproductive health is a set of priority activities that are implemented at the onset of every humanitarian emergency to prevent mortality, morbidity, and disability among crisis-affected populations. MISP activities should be expanded as soon as possible and should continue throughout protracted crises and recovery.^22^ Objective 4 of the MISP aims to prevent excess maternal and newborn morbidity and mortality, and one key element of this objective is to ensure the availability of lifesaving PAC in health centers and hospitals. Objective 5 of the MISP is to reduce unintended pregnancies by improving availability of contraceptive services, including contraceptive counseling and a wide range of contraceptive methods, and promoting community awareness of contraceptives.^23^ Despite recognition of the MISP as a global standard, it is not always implemented systematically, nor are all components always present after the onset of an emergency.^6^^,^^20^ The need for reproductive health services remains in crises; thus, access to PAC and the subsequent immediate delivery of quality voluntary contraceptive services should be ensured for all women, regardless of setting.

Quality PAC including both treatment of complications and immediate, on-site postabortion voluntary contraception, are essential in all settings, including acute emergencies. Investments in capacity building, supplies and infrastructure, community mobilization, and consistent monitoring make it possible for PAC to be provided in even the most challenging of settings.

## INTERVENTION

To address the issue of high maternal morbidity and mortality due to complications of induced abortion or miscarriage among women living in humanitarian settings, Save the Children began implementing PAC in 2012, including postabortion voluntary contraception, in diverse emergency settings. The current study focuses on 3 specific settings—Democratic Republic of the Congo (DRC), Somalia, and Yemen. In all these settings, the initial supply and demand for PAC was universally low because the services were typically relegated to referral hospitals where cost, transport, distance, provider competency, and supply availability were notable barriers. In addition, a great deal of community stigma existed around abortion, including seeking PAC.^24^ Further, many communities were unfamiliar with PAC in general, were not aware of danger signs, and did not know that such services existed, particularly at lower-level health facilities as opposed to exclusively within referral hospitals.

The current study focuses on PAC delivered by Save the Children in the DRC, Somalia, and Yemen.

**FIGURE 1:**
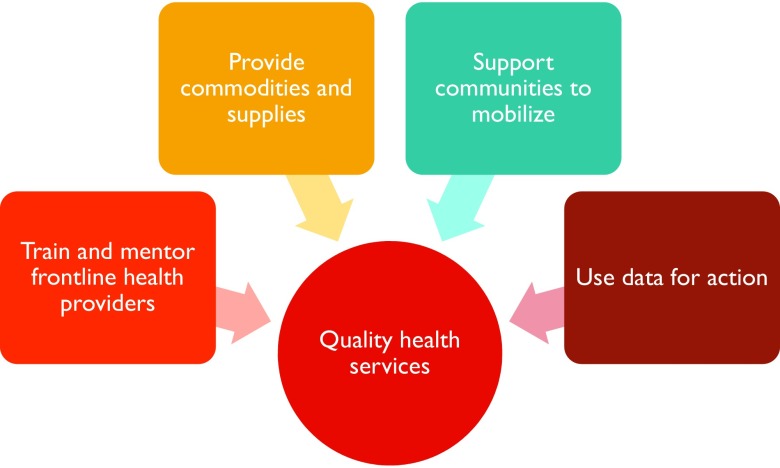
Save the Children Approach to Implementing Postabortion Care

Each country program applied a service approach based on *The Essential Elements of Postabortion Care* as developed by the PAC Consortium: community mobilization, strengthening provider counseling to identify and respond to women's emotional and physical health needs, treatment of abortion complications, provision of voluntary contraceptive services to help women prevent an unintended pregnancy or practice birth spacing, and referrals for any further health care required.^25^ Ideally, contraceptive counseling and services are provided as part of PAC by the same provider in the same location to ensure that opportunities for voluntary postabortion contraception are not lost through complicated referrals or the need for a woman to return to the facility at a later date. Save the Children implemented and upgraded PAC using a prioritized approach to ensure high-quality sexual and reproductive health services in humanitarian settings (Figure 1). This approach includes capacity building, assurance of supplies and infrastructure, community collaboration and mobilization, and consistent data management for ongoing monitoring, evaluation, and data use. Its goal is to improve the quality of PAC across facilities and develop greater understanding of the importance of PAC within communities so that women and girls can access comprehensive PAC without delay.

Save the Children implemented and upgraded PAC to ensure high-quality sexual and reproductive health services in humanitarian settings.

### Capacity Building

Capacity building focuses on improving the clinical competency of providers to provide a package of quality PAC. This package includes the ability to identify and diagnose complications due to abortion; conduct physical examination of the PAC client to determine treatment needed; provide surgical or medical treatment with pain management, infection prevention, and blood transfusion, where available; and provide contraceptive counseling and, if the client desires, a contraceptive method prior to discharge from the facility. To ensure that postabortion contraceptive methods were provided to all PAC clients who wanted them, all PAC clinical trainings included extensive focus on contraceptive counseling as well as the technical skills needed to provide a broad mix of contraceptive methods, including LARCs. Capacity building was organized through trainings, ongoing supportive supervision, and coaching from program and Ministry of Health (MOH) staff.

### Assurance of Supplies and Infrastructure

Supplies provided to supported health facilities included MVA kits, misoprostol for PAC, drugs for pain management, antibiotics, and materials for infection prevention and control. Medicines and equipment are monitored and maintained to ensure that there are no stock ruptures. Save the Children developed linkages with existing supply chains, including coordination with the United Nations Population Fund (UNFPA) and Ipas, to ensure that supplies and equipment are available even in constantly changing environments. Further, prepositioned stock is maintained in centralized warehouses to ensure that necessary PAC, contraceptive, and maternal health supplies can be deployed as rapidly as possible on request in even the most complex of situations. A key intervention to prevent the recurrence of unintended pregnancy is to ensure a broad contraceptive method mix.^19^ In some instances, new contraceptive methods were added to the existing method mix in partnership with the MOH to ensure that necessary approvals were in place and that these products could be easily added to existing supply chain mechanisms.

### Community Collaboration and Mobilization

To provide PAC, quality services must be in place; however, members of the community also need to know that they exist and be supportive of access. In order to provide information about PAC, community health workers conducted small group sessions to raise awareness of PAC availability, educate women and men on danger signs for when to seek care, and address any myths or misconceptions within surrounding communities. In many communities where abortion is taboo, it was important to present PAC as an essential part of maternal health care and a lifesaving service that is a critical part of a functioning health system. In addition to awareness campaigns within communities, key religious and secular leaders were involved in shaping the discourse and spreading key messages to their constituencies.

To provide PAC, quality services must be in place; however, members of the community also need to know that they exist.

### Data Management, Monitoring, and Evaluation

As the investments in PAC increased, including clinical training, assurance of supplies and equipment, and community mobilization activities, it was necessary to integrate functioning data management systems to ensure that access to PAC increased, postabortion complications were safely treated using MVA or misoprostol, and PAC clients could easily access voluntary contraception. To that end, each facility was equipped with a confidential register book to note the treatment and care provided to each PAC client and submitted aggregated data monthly to the program team and MOH. A health management information system was established to track PAC aggregated at the individual facility level and health zone, district, and national levels, where applicable. Program staff engaged in regular conversations around incoming data with providers and MOH representatives; thus, troubleshooting could occur collaboratively and in real time. Finally, register reviews were conducted annually to ensure that PAC clients represented diverse demographic groups and were provided with care appropriate to their symptoms and diagnosis.

### Country Contexts

#### Democratic Republic of the Congo

The humanitarian situation in the DRC is characterized by intercommunal violence, political unrest, and disease outbreaks resulting in an estimated 12.8 million people in need of humanitarian assistance and protection in 2019, which represents 10% of the total humanitarian caseload globally. This protracted humanitarian crisis has deteriorated significantly over the past years, and the country has the highest number of internally displaced people in Africa.^26^ The deepening crisis is most acute in the eastern part of the country, which includes North Kivu Province where Save the Children and the MOH have been working to strengthen the availability of PAC since 2011. At that time, most doctors had only been trained to use D&C for treatment of incomplete abortion at a referral-level facility, while midwives and nurses were not trained or authorized to provide PAC at all. The project is now working in 29 health facilities including 1 hospital and 4 referral health centers covering 5 health zones. MOH staff and project supervisors were trained as PAC trainers on methods of treatment (MVA and misoprostol) and postabortion contraception, inclusive of short- and long-acting contraception. These skills were then cascaded to midwives and nurses to provide PAC at a primary health level. Following the training, Save the Children and MOH supervisors jointly conducted supportive supervision and coaching for trained providers using a range of standardized tools and checklists to assess progress and provide on-the-job training where needed to improve the quality of PAC.

#### Somalia

The humanitarian crisis in Somalia is the longest continuous crisis in the world and is characterized by complex factors such as famine, drought, conflict, disease outbreaks, extreme poverty, and terrorism. An estimated 2.2 million Somalis are internally displaced by conflict and drought and constitute 40% of those in need of assistance within the country.^27^ In partnership with the MOH, Save the Children has been implementing a PAC program in the Karkaar region of Puntland since 2012 in 1 hospital and 9 primary health centers, improving the coverage and scale of service provision for Karkaar's population. Save the Children continues to be the only organization supporting the provision of PAC in Puntland State of Somalia. Facility infrastructures have been modified as required, to ensure that sufficient space and privacy are available for counseling and procedures. Each site has at least 2 trained health workers in place who offer PAC including MVA, misoprostol, and a full range of modern contraceptive methods, including LARCs and short-acting methods. Supportive supervision of health workers is conducted in collaboration with the regional health office using standardized checklists to monitor the quality of service provision. UNFPA and Save the Children work together to ensure the provision of required PAC supplies and commodities and support as necessary to ensure stock-outs do not occur and services remain available at all times.

In addition to physical and security barriers to accessing care, shame, stigma, and gender norms can further impede a woman from accessing PAC at a facility. The current phase of the program includes a strong community element, which aims to raise awareness about the importance of seeking PAC. A 3-month community PAC campaign was carried out in 2017 to increase the knowledge and acceptance of PAC in the local community. Routine facility data showed an increase in PAC client numbers that correlated with the timing of the campaign. Additionally, the team used the primary telecommunications provider, GOLIS, to disseminate weekly text messages on PAC, specifically on first-trimester complications, the need to seek medical treatment, and postabortion contraception, to 40,000 people in Karkaar region.

The program in Somalia includes a strong community element to raise awareness about PAC.

#### Yemen

The conflict and crisis in Yemen have been escalating since 2015, with more than 22 million people, including 11 million women and girls, in need of urgent humanitarian aid.^28^ Only a third to a half of health facilities are still functioning, exacerbating the long-standing barriers that Yemeni women already faced in accessing reproductive health services.^28^^,^^29^ The PAC program in Yemen has been implemented through 16 health facilities managed by the Ministry of Planning and Public Health in the Hodeida and Lahj governorates since 2013. Four hospitals and 12 primary health centers were supported to establish PAC for the first time. Discussions with governorate and district health offices allowed the establishment of PAC at the primary health center level, integrated with other reproductive health services. Following authorization, Save the Children rehabilitated the health centers to create PAC procedure rooms and provided the necessary medical supplies, equipment, and medicines. Clinical providers, particularly the midwife cadre, were trained in providing misoprostol for treatment of incomplete abortion, MVA, and postabortion contraception. Supportive supervision was provided to these health facilities to ensure that infection prevention standards were met and that PAC and voluntary postabortion contraceptive services, including LARCs, were provided according to WHO standards. Specific PAC registers and client forms were introduced and midwives were trained to collect and review data to inform their service provision at respective health facilities. In spite of the worsening situation in the country since 2015, the program has seen a steady flow of clients requesting PAC.

## METHODS

To evaluate the overall impact of a comprehensive PAC program approach on improved quality of treatment and postabortion contraception, we analyzed service delivery data from each of the 3 countries from the inception of their program through 2017. The DRC program began in 2011 and data collection commenced in 2012. The Somalia program was launched in 2012 and the Yemen program in 2013. The indicators evaluated for this article included the overall number of PAC clients, mode of treatment, the proportion of PAC clients who chose a method of contraception prior to leaving the facility, and the contraceptive method mix among those PAC clients since demand varies across methods.^21^^,^^30^

Changes in service delivery trends over time were observed for each of the indicators. Tests of association were performed to assess the significance of changes in treatment with D&C as a proportion of all PAC cases and changes in contraceptive uptake among all PAC clients.

To better understand best practices for raising awareness around PAC at a community level, we analyzed qualitative program data including evaluations of community mobilization activities as well as informal interviews with community members and leaders. PAC community awareness campaigns were conducted in the DRC in mid-2016 with refresher trainings in mid-2017; in Somalia from January to April 2017; and in Yemen in mid-2018.

## RESULTS

### Service Delivery Data

**FIGURE 2:**
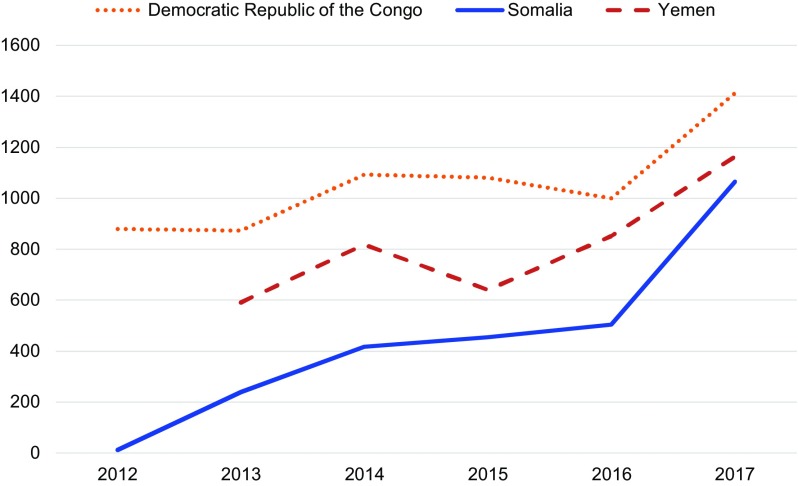
Total Number of Postabortion Care Clients, Democratic Republic of the Congo, Somalia, and Yemen, 2012–2017

The number of overall PAC clients increased over time in all 3 countries (Figure 2). In the DRC, there were 812 PAC clients in 2012 compared with 1,412 PAC clients in 2017. In Somalia, PAC clients increased from 11 in 2012 to 1,065 in 2017. In Yemen, the number of PAC clients rose from 590 in 2013 to 1,163 in 2017. The number of PAC clients increased due to improved service availability and increased emphasis on improving community messages around the importance and availability of PAC. We observed an increase in PAC clients in the DRC and Somalia that coincided with their PAC-specific community awareness efforts in late 2016 and early 2017. However, in Yemen, where PAC-specific community awareness campaigns were not conducted during the 2013–2017 period, the total number of PAC clients increased, indicating that improvements in provider capacity and service availability are also instrumental in increasing demand for PAC.

The number of overall PAC clients increased over time in all 3 countries.

**FIGURE 3:**
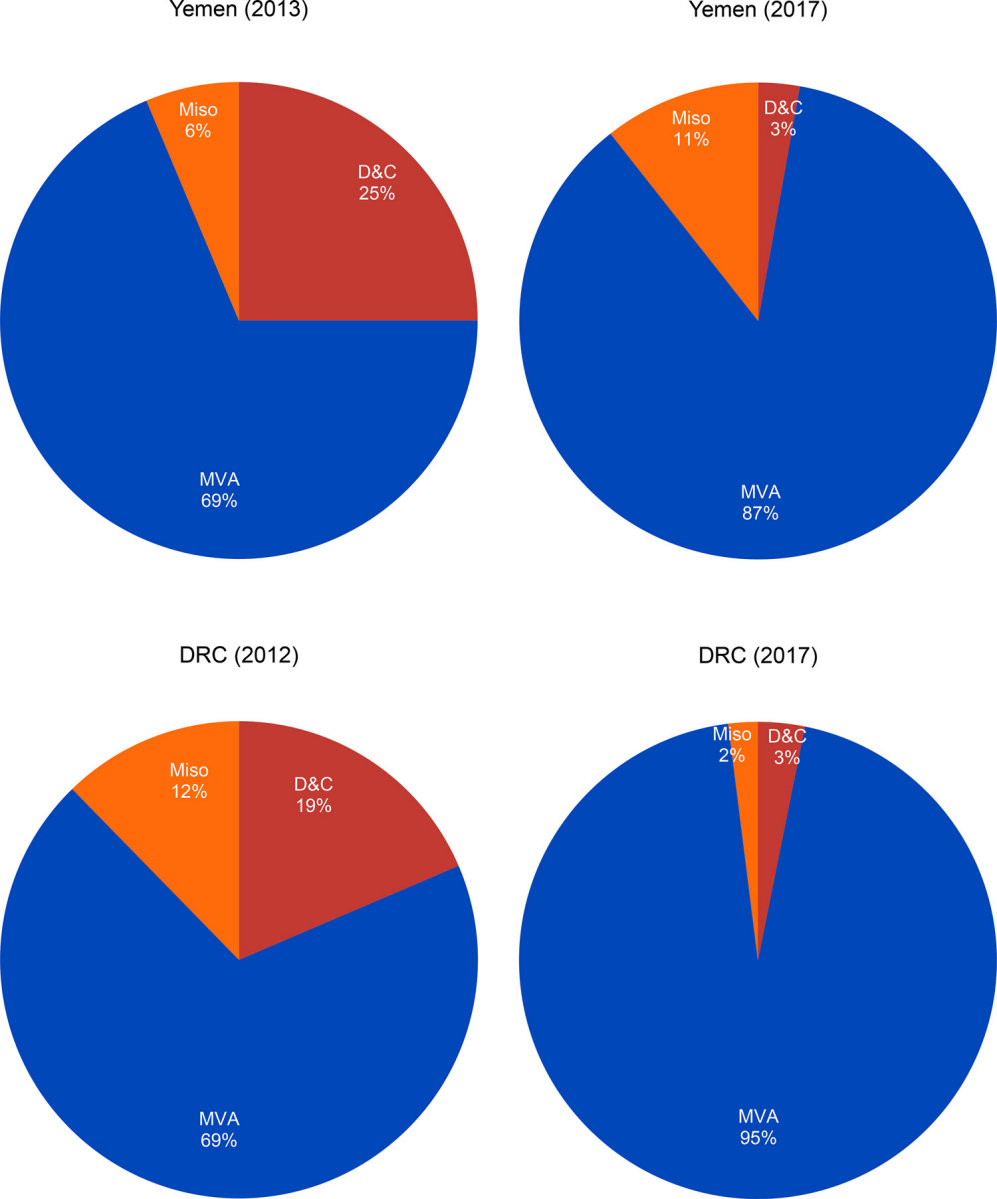
Method of Treatment Among Postabortion Clients in the DRC and Yemen, First Year of Program Implementation vs. 2017 Abbreviations: D&C, dilation and curettage; DRC, Democratic Republic of the Congo; MVA, manual vacuum aspiration; Miso, misoprostol.

**FIGURE 4:**
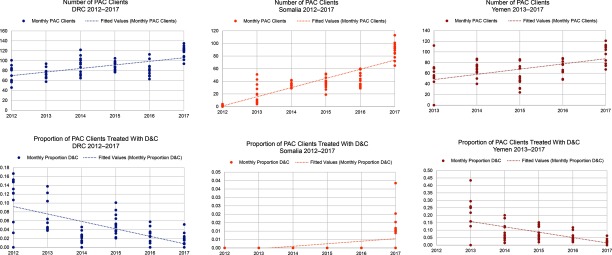
Overall Number of Postabortion Care Clients and Proportion Treated With Dilation and Curettage in the DRC, Somalia, and Yemen, 2012/2013–2017 Abbreviations: D&C, dilation and curettage; DRC, Democratic Republic of the Congo.

Both the DRC and Yemen had a noteworthy reduction in the use of D&C as a method of treatment among all cases of PAC in 2017 compared with their first year of program implementation (*P*<.001). In the DRC in 2012, 19% of all PAC clients requiring evacuation received D&C as treatment, whereas in 2017 the percentage was reduced to 3% (Figure 3). When the Yemen program commenced in 2013, 25% of all PAC clients were treated with D&C. By 2017, D&C was reduced to 3% of all PAC clients (Figure 3). In Somalia between 2012 and 2017, the percentage of PAC clients treated with D&C actually increased from 0% to 1%; however, the total number of PAC clients increased from 11 in 2012 to 1,065 in 2017. Monthly trends in the overall number of PAC clients and the proportion treated with D&C are displayed in Figure 4. Each circle represents the aggregated monthly total of all supported facilities within the country program area.

**FIGURE 5:**
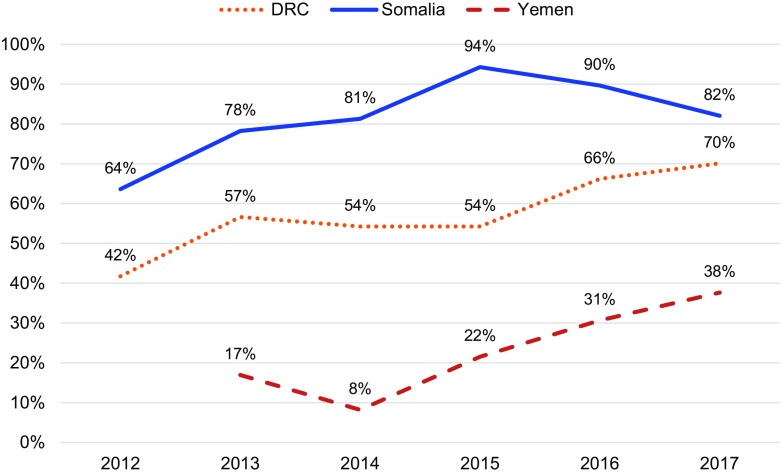
Percentage of Postabortion Care Clients Voluntarily Choosing Contraception Prior to Discharge in the DRC, Somalia, and Yemen, 2012–2017 Abbreviation: DRC, Democratic Republic of the Congo.

**FIGURE 6:**
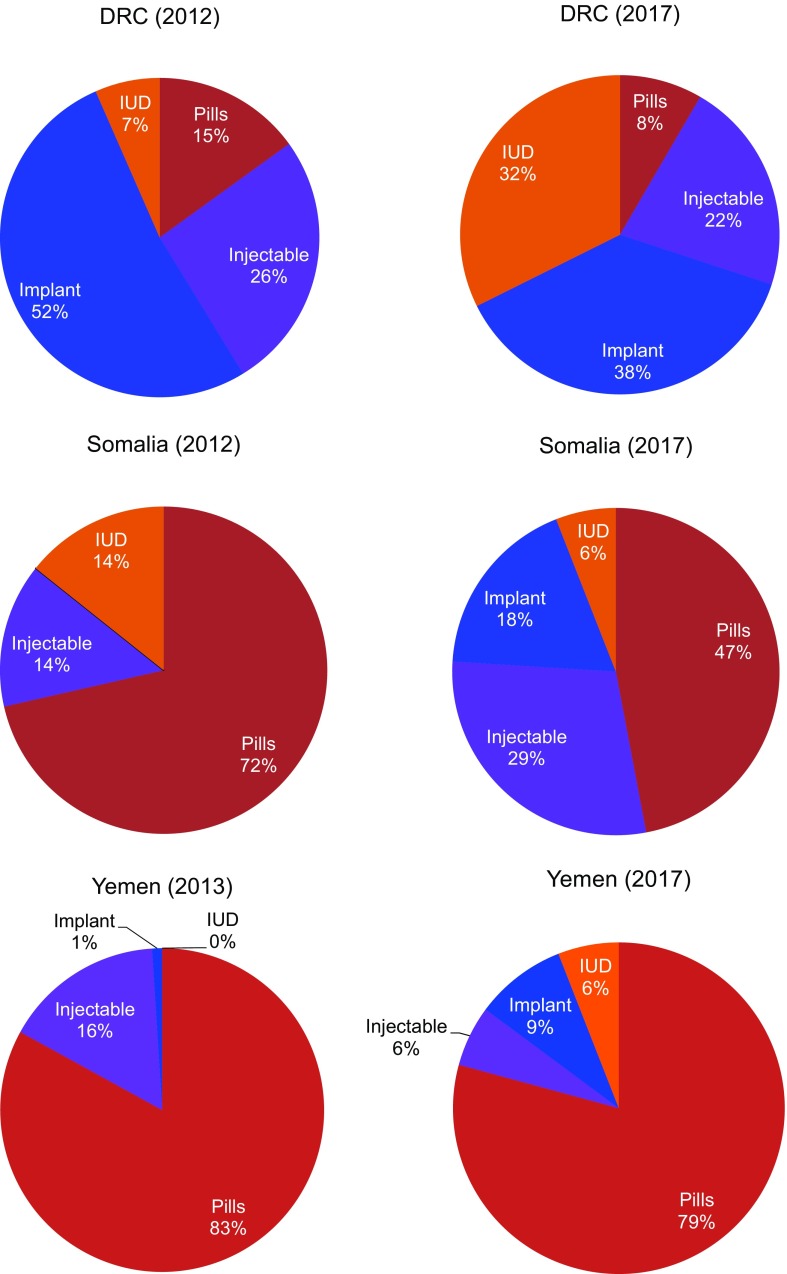
Postabortion Care Contraceptive Method Mix in the DRC, Somalia, and Yemen, 2012/2013–2017 Abbreviation: DRC, Democratic Republic of the Congo; IUD, intrauterine device.

**FIGURE 7:**
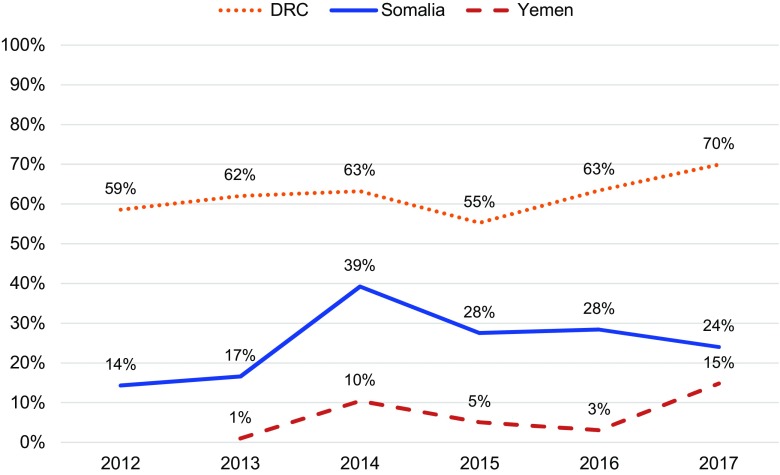
Percentage of PAC Clients Choosing LARCs Among Those Choosing Contraception, DRC, Somalia, and Yemen, 2012–2017 Abbreviations: DRC, Democratic Republic of the Congo; PAC, postabortion care; LARCs, long-acting reversible contraceptives.

In all 3 countries, the proportion of women who chose a method of contraception after treatment for abortion complications increased (Figure 5). This change was significant in the DRC (*P*<.001) and Yemen (*P*=.002), but not in Somalia where postabortion contraception uptake has been relatively high since the inception of the program. Further, all settings saw shifts in method mix over time (Figure 6), which included an increase in the proportion of women who chose LARCs after treatment for abortion complications (Figure 7). Again, this increase was significant in the DRC (*P*=.02) and Yemen (*P*=.004), but not in Somalia. Although a shift occurred toward a greater proportion of women and girls choosing a LARC method after treatment for abortion complications, this trend varied over time.

In all 3 countries, the proportion of women who chose a method of contraception after abortion increased.

**FIGURE 8:**
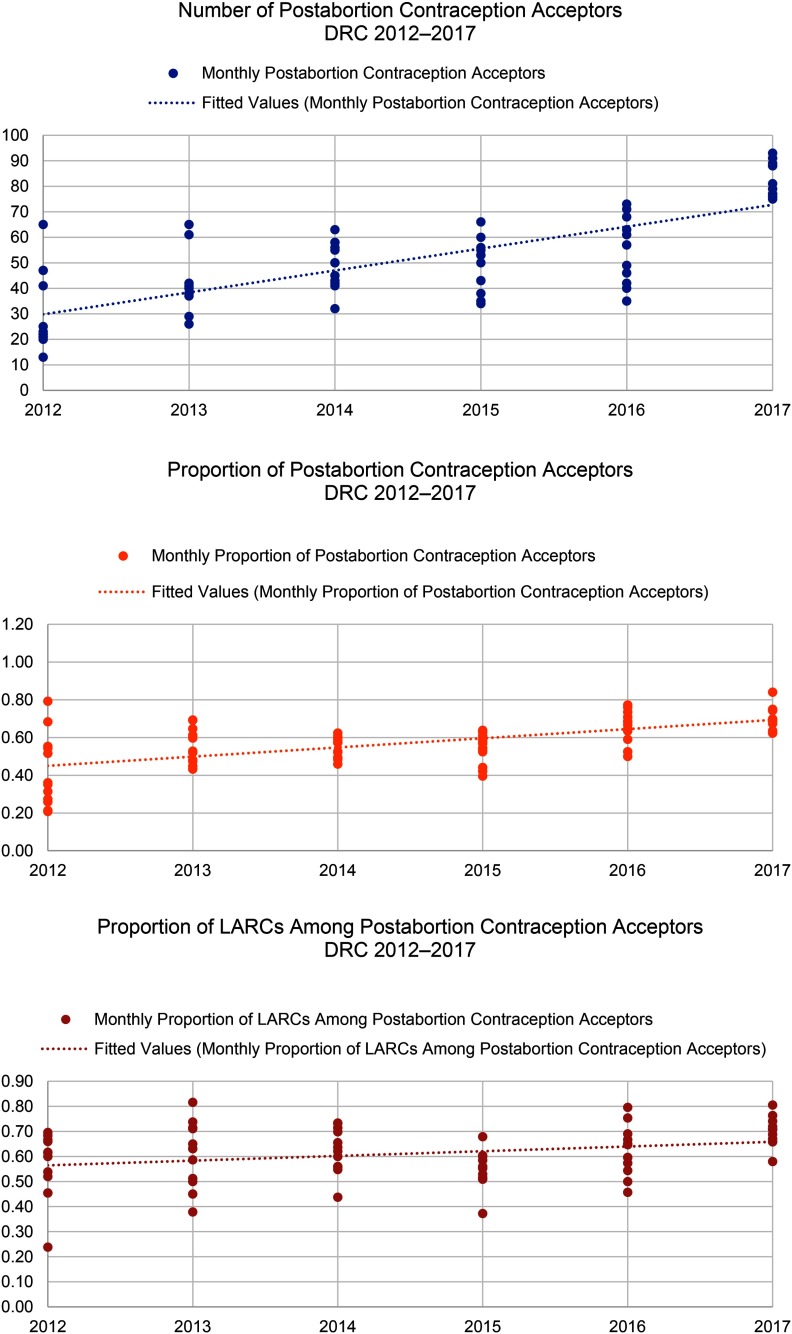
Trends in Postabortion Care Contraception in the DRC, 2012–2017 Abbreviations: DRC, Democratic Republic of the Congo; LARCs, long-acting reversible contraceptives.

**FIGURE 9:**
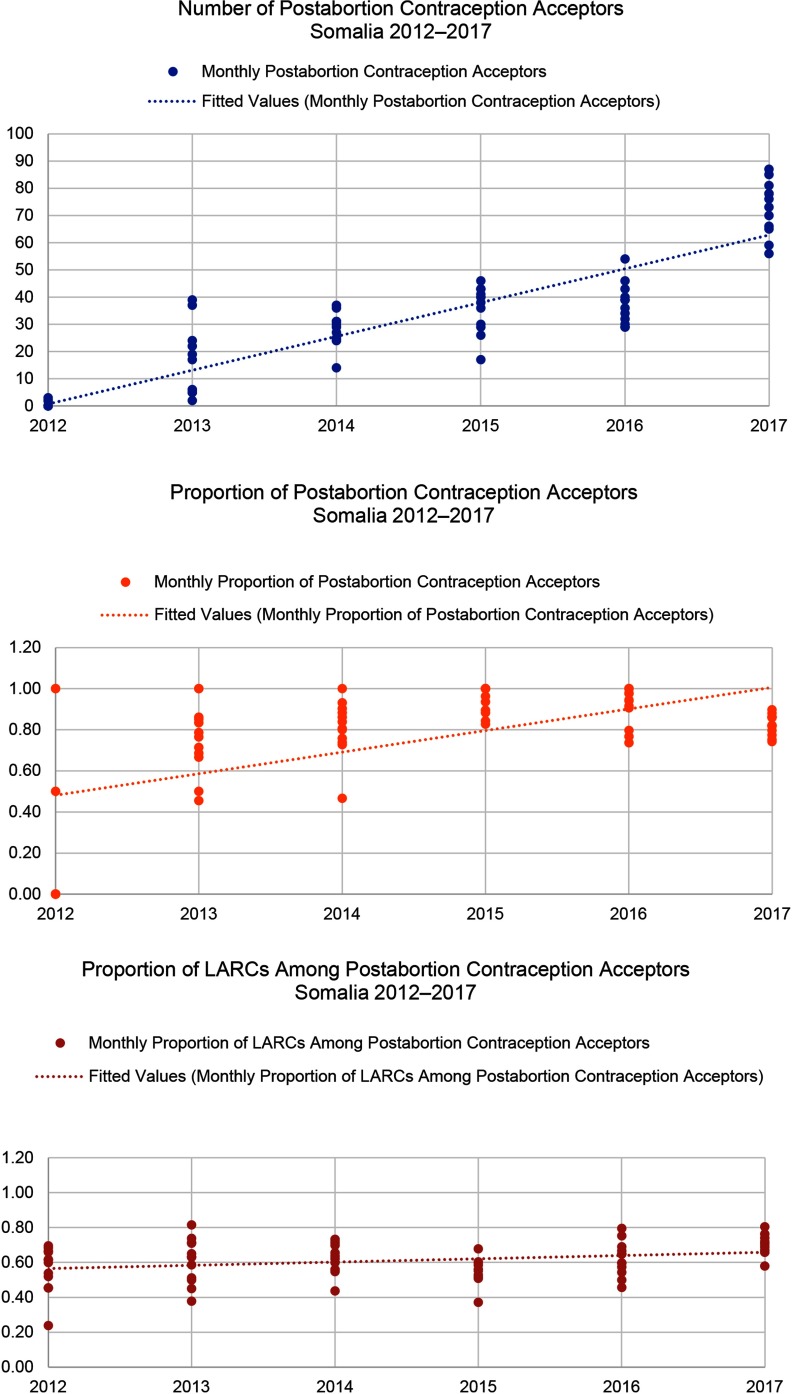
Trends in Postabortion Care Contraception in Somalia, 2012–2017 Abbreviation: LARCs, long-acting reversible contraceptives.

**FIGURE 10:**
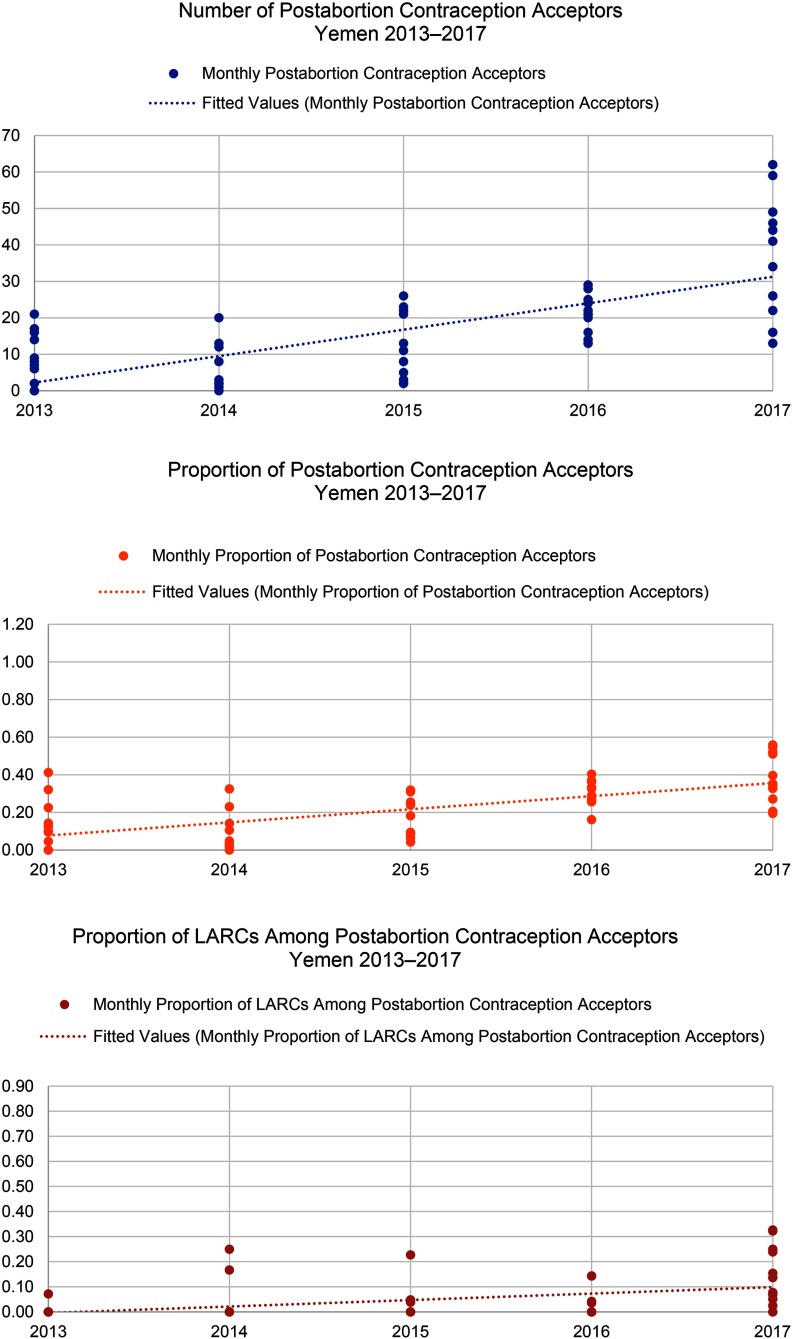
Trends in Postabortion Care Contraception in Yemen, 2013–2017 Abbreviation: LARCs, long-acting reversible contraceptives.

When Save the Children began supporting health facilities to provide PAC in the DRC in 2012, only 42% of all PAC clients adopted a method of contraception before discharge. By 2017, the percentage had increased to 70%. The selection of LARCs as a percentage of postabortion contraception increased from 59% in 2012 to 70% in 2017. Somalia had notable increases in demand for postabortion contraception, with the percentage of all PAC clients choosing a contraceptive method increasing from 64% in 2012 to 82% in 2017 and the percentage of all PAC clients choosing a LARC method increasing from 14% in 2012 to 24% in 2017. Finally, in Yemen, where the health system has been constrained due to severe conflict, the percentage of PAC clients choosing a contraceptive method rose from 17% in 2013 to 38% in 2017. Though LARCs were not a substantial part of the contraceptive method mix in Yemen, the percentage of total PAC clients choosing an intrauterine device or implant also increased, from 1% in 2013 to 15% in 2017. Monthly trends in the number of overall postabortion contraception clients, proportion choosing a method of contraception, and proportion of postabortion contraceptive users choosing LARCs are displayed in Figure 8 for the DRC, Figure 9 for Somalia, and Figure 10 for Yemen; each circle represents the aggregated monthly total of all supported facilities within the country program area.

### Qualitative Program Data

In addition to ensuring clinical training, supplies, and ongoing data use, supporting communities to mobilize around the sharing of sexual and reproductive health information and services is essential. The analysis of community mobilization reports found that many program teams were accustomed to generating community awareness and demand around voluntary contraception, but identifying best practices around PAC messaging at a community level was unfamiliar. To this end, each country program developed contraception and PAC outreach strategies that included specific target populations, key messages, and channels of communication. Using these strategies, teams developed activities and materials to raise awareness of PAC, teach communities to identify and facilitate referral for life-threatening postabortion complications, and to educate communities on the value and availability of voluntary postabortion contraception. Refresher trainings for community health workers and volunteers were conducted throughout the period to ensure that they had the necessary skills to deliver key PAC messages through a variety of learning modalities.

Despite differences in geography, language, and culture, the qualitative program data from the DRC, Somalia, and Yemen suggested common best practices as well as challenges when raising awareness around PAC in communities that are often unfamiliar with the topic and are simultaneously experiencing instability and population movement due to conflict. Before beginning work directly in the community, all programs first convened secular and religious community leaders in a day-long meeting to ensure that they understood the importance of PAC, had time to ask questions, and were supportive of the campaigns. All programs found that information sharing from health care providers and members of the community were useful in conveying information about the importance of supporting PAC. In Yemen, a woman told her story about having had multiple abortions and receiving information from a community health volunteer about voluntary contraception and the importance of spacing intervals between miscarriages and pregnancy. She went to a health facility, sought counseling, chose a contraceptive method, and after waiting a sufficient time, was able to become pregnant and now has a healthy child. In the DRC, a 28-year-old woman noted the important role of community health workers:

It's the [community health workers] who teach us, even those here, these women, they often pass through the neighborhood teaching us, if there's a miscarriage, you must hurry to the hospital and if the stomach really hurts when pregnant, you must hurry to the hospital to be treated.

Despite differences in geography, language, and culture, the qualitative program data from the 3 countries suggested common best practices as well as challenges.

In Somalia, a female doctor delivered lectures on abortion, danger signs that threaten the life of mothers during pregnancy, available services, and when to seek help at each campaign event. The audience was actively engaged and many asked questions, especially regarding the link between PAC and contraception. The creation of dramas and songs that conveyed information about PAC proved popular in all 3 locations and were useful in attracting a broad audience through entertaining vignettes. Information was also conveyed on the radio, via mobile phone in Somalia, and through leaflets and posters explaining what to do when you recognize danger signs in the community. A 25-year-old woman from the DRC highlighted the importance of sharing key PAC messages through a variety of modalities:

No one advised me [on PAC], I just heard it on the radio that if you have this type of problem, you must go to the hospital.

Despite reaching large audiences, all programs aimed to reach more men in the future with PAC messaging. In late 2016, the DRC program piloted an effort to engage men who were satisfied with PAC received by their female partner. These clients were trained and conducted a participatory review of the community mobilization strategy, offering inputs. Satisfied male advocates were identified as important for initiating conversations regarding PAC. The most referrals for PAC were from health care providers, community health workers, and male advocates in the community. Due to the success of this pilot, the DRC program scaled up this model of identifying and training satisfied male advocates in the community to spread important PAC messages.

In Somalia and Yemen, many men said they felt PAC was a women's issue and did not think the sessions would be relevant to them, even though men are very important in enabling access to PAC and facilitating referrals. Although these PAC awareness campaigns were effective in reaching a large number of people, they were very time consuming. Program staff indicated that they would like to follow up with participants over time to determine what messages were the most salient and which were most closely linked to changes in individual and community behavior. Despite challenges, however, community awareness campaigns were linked to increases in PAC clients at health facilities and, over time, increased uptake of postabortion contraception.

## DISCUSSION

Although complications from abortion are documented causes of excess morbidity and mortality among women living in humanitarian contexts, PAC is often only available at hospital-level facilities and only with treatment via D&C, further impeding access in already constrained areas. Increasing availability and improving the clinical approach by supporting primary health facilities and midwives to provide MVA, misoprostol, and voluntary postabortion contraceptive services expands access and reduces morbidity and mortality among women in need of PAC in humanitarian settings.

Increasing the availability of PAC and improving the clinical approach reduces morbidity and mortality among women in humanitarian settings.

These data demonstrate that providers can effectively shift away from D&C toward MVA and misoprostol and that voluntary contraceptive uptake for PAC clients can increase substantially, even in settings where the use of postabortion contraception is often stigmatized. A multifaceted approach was taken in each country to ensure that quality PAC was implemented in an effective and respectful manner. The important link between postabortion contraception and the avoidance of future unintended pregnancies was understood by PAC clients, as evidenced by increased postabortion voluntary contraception uptake. This approach focused on health worker capacity building, continuous quality improvement on supplies and service delivery, consistent collaboration with the local health authorities, sustained community engagement, and strengthened health information monitoring systems.

The findings show that MVA eclipses misoprostol in the treatment of abortion complications. In many countries where Save the Children works in humanitarian emergencies, misoprostol is not on the national-level list of approved drugs for the treatment of postabortion complications or has only recently become a part of approved treatments, or its administration is limited by required supervision from medical doctors. It therefore takes time to shift the practice toward misoprostol for PAC, despite providers receiving training on both techniques. Further, the data are from public facilities only and do not consider women who seek misoprostol at a pharmacy or private clinic.

The results of this program model suggest the feasibility and acceptability of midlevel health care workers providing PAC at primary health level facilities, and the findings should be used to advocate for necessary policy shifts, including task sharing, particularly in countries affected by conflict where access to health care is already constrained. Midlevel providers should be supported through preservice and in-service training on the provision of misoprostol, MVA, and voluntary contraception for PAC clients. Procurement and supplies for primary-level health facilities should include misoprostol, MVA kits, contraception methods including LARCs, and necessary infection prevention materials. Health information systems should incorporate relevant PAC indicators to monitor availability and accessibility of PAC throughout an emergency.

Based on the findings from community mobilization evaluations, we see a need to create demand for and understanding of PAC, which constitutes a lifesaving and essential element of a quality health system. In humanitarian settings where systems have been disrupted, many people were not aware that PAC existed, especially outside of referral hospitals. Further, messages around PAC need to be conveyed in a variety of ways in order to reach a broad audience that includes community leaders, men, women, and young people. Although raising community awareness is a common element of contraceptive service provision, this sort of messaging around PAC was new to many of the project locales and required new and creative ways of thinking about community-level buy-in and education.

In humanitarian settings, many people were not aware that PAC existed.

Overall, this particular model of providing PAC is intensive and comprehensive. In many instances, LARCs were introduced into the method mix at the commencement of this program and had varying levels of uptake. Each of the featured countries—the DRC, Somalia, and Yemen—had shifts in overall contraceptive method mix as well as postabortion contraceptive method mix. In the DRC, implants have been very popular since their introduction, with intrauterine devices slowly increasing in popularity. In Somalia and Yemen, short-acting methods of contraception were the largest proportion of the respective method mixes with LARC uptake increasing slowly. Shifting a program to facilitate greater availability of and access to LARCs requires skilled counseling and consistent supplies. Because both were a feature of this program, we have seen shifts over time toward greater LARC representation in the method mix of all 3 countries, but at different rates.

Funding for PAC often comes through funding for broader emergency obstetric and newborn care (EmONC) programs within humanitarian responses. This program model had a clear focus on PAC within clinical trainings, supplies, supervision, and community mobilization. PAC was able to be offered at primary-level facilities, even when the facilities did not provide other EmONC services, allowing increased access. Having dedicated funding geared specifically toward quality PAC and contraception allows for true improvements in provider skill, provision of good services, shifts in perceptions, and, ultimately, a better overall health system for women, families, and communities.

### Limitations

The study design was retrospective in nature. While aggregated data on the number of PAC clients each month at each facility were collected monthly throughout program implementation, data from pre-implementation were not available for comparison. Aggregated facility data only allowed us to examine certain indicators such as the overall number of clients and the proportion who voluntarily adopt postabortion contraception. Demographics of the women accessing voluntary contraceptive services and estimates of remaining unmet need could not be evaluated. Neither qualitative information from trained providers on their experiences nor survey information from PAC clients on satisfaction with the services provided were available for this analysis.

## CONCLUSION

This program implementation model has been effective at expanding access in these 3 settings, which are much like many other humanitarian settings. However, there remains a high unmet need for quality PAC throughout humanitarian environments. More resources must be committed to further expand the provision of quality PAC in crisis-affected countries in order to treat postabortion complications, ensure effective voluntary contraceptive counseling and provision, contribute to the overall reduction in maternal mortality, and serve PAC client needs globally.

More resources must be committed to further expand the provision of quality PAC in crisis-affected countries.

## Notes

**Peer Reviewed**

**Funding:** Anonymous private foundation.
